# Global Sensitivity Analysis of Societal Resilience Using Shapley Values and Polynomial Chaos Expansion

**DOI:** 10.1111/risa.70295

**Published:** 2026-06-28

**Authors:** Elias Montanari, Ioannis Galariotis, Andrea Cappannini, Thierry Mara, Christine Redecker

**Affiliations:** ^1^ Joint Research Center Ispra Italy; ^2^ Unisystems S.A, Luxembourg; ^3^ Unisystems S.A Luxembourg; ^4^ Universite’ de la Reunion, France Reunion France

**Keywords:** European Union, polycrisis, resilience modeling, risk assessment, sensitivity analysis, Shapley value, societal resilience

## Abstract

Societies worldwide face increasingly complex and interconnected crises that challenge their capacity for resilience. Assessing which structural indicators are most strongly associated with resilience scores requires quantitative methods capable of handling interdependencies, nonlinearities, and limited sample sizes. This study applies established global sensitivity analysis tools within an empirical resilience setting characterized by correlated structural indicators and small cross‐national datasets. Using 124 indicators from the Joint Research Centre Resilience Dashboards, we analyze model‐based structural correlates of societal resilience across EU Member States using the Lloyd's Register Foundation World Risk Poll Resilience Index for 2021 and 2023. Following univariate Pearson correlation screening and Random Forest stability selection, Shapley‐based variance decomposition is computed on a Polynomial Chaos Expansion (PCE) surrogate, enabling equitable attribution under correlated inputs. For 2023, the results indicate a concentrated attribution structure, with a small core of indicators accounting for most explained variance (*R* ^2^ = 0.85). Active citizenship has the largest model‐based contribution, followed by antimicrobial resistance and years of life lost due to PM_2_._5_ exposure, suggesting strong associations with social capital and environmental health under sustained polycrisis conditions. Environmental innovation, ecological conditions, and macroeconomic buffering form a secondary tier of contributors. By contrast, the 2021 model exhibits a more diffuse attribution profile (*R* ^2^ = 0.82), with resilience scores associated more evenly with active citizenship, adult competences, digital readiness, and labor‐market adjustment. Leave‐one‐out validation suggests that these attribution patterns are not dominated by single‐country exclusions, although uncertainty remains substantial in a small cross‐sectional sample. Overall, the comparison indicates a shift in the fitted attribution structure from broader recovery‐oriented human‐capital and digital indicators in 2021 toward more concentrated structural contributions linked to social and environmental conditions in 2023, offering a transparent methodological framework for comparative resilience analysis in small‐sample policy settings.

## 1 Introduction

The European Union (EU) has faced multiple overlapping crises in recent years, including the COVID‐19 pandemic, Russia's war of aggression against Ukraine, climate‐related disasters, and economic disruptions (European Commission 2023; Tooze 2022). These exemplify the interlinked nature of crisis dynamics in a polycrisis context, where simultaneous shocks interact such that the overall impact exceeds the sum of individual events. Exploring why some countries, communities, and systems appear better able to absorb and recover from shocks requires methodological approaches capable of assessing which structural factors are associated with resilience capacity.

Numerous frameworks monitor resilience, including the European Commission's Joint Research Centre (JRC) Resilience Dashboards (Benczur et al. 2023a) and the Lloyd's Register Foundation (LRF) World Risk Poll (Lloyd's Register Foundation 2022, 2024). Whilst these frameworks are useful for assessing country performance, they do not by themselves show how different social, economic, environmental, and institutional capacities are associated with resilience outcomes.

Traditional statistical methods, including regression, principal‐component analyses, and composite‐index approaches, assume linear and independent effects, an assumption rarely satisfied in complex systems where interdependencies and feedback loops prevail (Folke et al. 2010; Scheffer et al. 2001; Walker et al. 2004). Resilience is emergent: it arises from the dynamic interaction of social, economic, environmental, and institutional subsystems. Moreover, high multicollinearity among structural indicators prevents equitable distribution of explanatory power (Kline 2017; Belsley et al. 1980; O'Brien 2007).

Sensitivity analysis (SA) provides a principled framework for addressing these challenges. SA examines how uncertainty or variability in model inputs propagates to uncertainty in model outputs, thereby allowing the analyst to rank inputs by their relative influence on system‐level behavior (Saltelli et al. 2008; Helton et al. 2006; Borgonovo 2017). Classical variance‐based methods, notably Sobol’ indices, decompose output variance into first‐order and higher‐order components (Sobol’ 2001; Saltelli et al. 2010). However, Sobol’‐type decompositions assume input independence, an assumption violated in socioeconomic indicator systems where structural correlations among inputs are pervasive (Borgonovo et al. 2012; Owen and Prieur 2017). Shapley‐value‐based SA, rooted in cooperative game theory (Shapley 1953), overcomes this limitation by providing a uniquely fair and efficient attribution of output variance to correlated inputs (Owen 2014). This makes it particularly suited to cross‐national resilience modeling.

Observed cross‐sectional data on 27 EU Member States are drawn from two harmonized databases. The JRC Resilience Dashboards supply 124 structural capacity indicators as independent variables; the LRF World Risk Poll Resilience Index supplies a perception‐based societal resilience score as the dependent variable. No physical or simulation model governs the data‐generating process. Instead, the analysis learns the input–output mapping from data via a Polynomial Chaos Expansion (PCE) surrogate, which is then used to compute Shapley‐based variance attributions. In this respect, the analysis is conducted in a given‐data setting, where sensitivity attribution is performed after estimating a surrogate representation of the empirical input–output relationship rather than propagating uncertainty through a predefined mechanistic model.

To address this methodological challenge, this study introduces a two‐stage analytical framework. First, univariate Pearson correlation screening, followed by Random Forest (RF)‐based stability selection, reduces the original 124‐indicator input space to a parsimonious and stable subset of predictors. Second, a Shapley‐based global sensitivity analysis (GSA) implemented on a PCE surrogate decomposes the explained variance into additive contributions attributed to each selected indicator, consistent with the efficiency, symmetry, and marginality axioms of Shapley decomposition. The PCE surrogate is preferred over an RF surrogate for Shapley estimation because variance decomposition from PCE coefficients can be derived analytically and computed efficiently once the surrogate is fitted, whereas RF‐based Shapley estimation typically requires additional approximation procedures (e.g., Bénard et al., 2022) or extensive simulation. Moreover, PCE provides an explicit orthogonal functional basis from which moment‐based sensitivity indices can be directly obtained (Mara and Becker, 2021; Iooss and Prieur, 2019). The framework is applied separately to 2021 (*R* ^2^ = 0.82) and 2023 (*R* ^2^ = 0.85) to examine how the relative structure of resilience‐related associations differs across two crisis‐related policy contexts. The comparison is intended as an analytical contrast between two resilience configurations rather than as a longitudinal trend analysis.

Rather than proposing a new estimator, the article demonstrates that Shapley‐based GSA can remain analytically informative in an associational, given‐data setting where classical attribution methods become unstable: small samples, strong predictor dependence, heterogeneous structural indicators, and absence of a predefined simulation model.

Beyond its methodological focus, this framework can support policy discussion by quantifying model‐based contributions of structural indicators to societal resilience scores in Europe. It advances GSA beyond its traditional engineering and physical‐science applications, extending it to complex societal systems and operationalizing principles of explainable risk assessment as promoted by the Sensitivity Analysis at 30 initiative.

The remainder of the article is organized as follows: Section 2 formulates research objectives. Section 3 reviews theoretical and empirical approaches to resilience and SA. Section 4 describes data and methodology, including the Shapley framework. Section 5 presents results for 2021 and 2023. Section 6 reports robustness and validation tests. Section 7 discusses findings in light of resilience theory and EU policy frameworks, and Section 8 concludes with limitations and implications for future research.

## 2 Research Questions and Objectives

Focusing on the European context, this study addresses the following research question: given the multidimensional and interdependent nature of societal resilience, how can we quantify and equitably distribute the contribution of structural indicators to resilience outcomes across the EU? To answer this question, we adopt a two‐stage analytical framework within GSA applied to societal resilience, pursuing three interrelated objectives.

1. *To identify robust model‐based correlates of societal resilience*: An initial prescreening stage employs univariate Pearson correlation tests to identify indicators exhibiting statistically significant associations with resilience outcomes (*p* ≤ 0.05) across EU Member States. Indicator robustness and nonredundancy are subsequently strengthened through RF‐based stability selection, which prioritizes variables showing stable predictive importance under correlated inputs and limited sample size (*n* = 27).
2. *To decompose model explanatory power into interpretable feature contributions*: Using a Shapley‐based SA framework, we decompose the explanatory capacity of a PCE regression metamodel fitted to the screened indicators into additive contributions attributable to each structural indicator. The Shapley‐based feature contribution of indicator *i* is defined as the Shapley value *φ_i_*, representing the average marginal contribution of indicator *i* to model performance across all possible subsets of the selected indicator set, as formalized in Section 4.2.5. PCE is used as the surrogate model because its polynomial coefficient structure permits analytical variance decomposition and efficient Shapley estimation under correlated inputs (Mara and Becker 2021; Iooss and Prieur 2019).
3. *To assess robustness and generalizability across countries and years*: Leave‐one‐out (LOO) SA is employed to evaluate whether estimated Shapley‐based feature contributions remain stable when individual countries are excluded. This allows us to distinguish systemic European‐level resilience associations from country‐specific patterns and to compare resilience structures across the two analytical contexts of 2021 and 2023.

## 3 Literature Review

### 3.1 Conceptual Background: Resilience as a System Property

Resilience has evolved from an ecological concept into a central organizing principle of risk governance and systems science. The United Nations defines resilience as “the ability of a system, community or society exposed to hazards to resist, absorb, accommodate, adapt to, transform and recover from the effects of a hazard in a timely and efficient manner” (2016, 21). Within the EU policy context, resilience underpins frameworks such as the Union Civil Protection Mechanism, the Strategic Compass for security and defense (Council of the European Union 2022), and the JRC Resilience Dashboards (Benczur et al. 2023a, 2023b).

In the academic literature, resilience is conceptualized as a multilevel, multidomain system property (Folke et al. 2010; Davoudi et al. 2012; Meerow et al. 2016). The concept has been operationalized across scales from individual psychological resilience (Luthar et al. 2000) to community resilience (Norris et al. 2008), reflecting its status as a “boundary object” bridging disciplinary perspectives (Brand and Jax 2006). This multidimensionality implies that resilience cannot be reduced to a single variable or index: it is an emergent outcome of interactions among subsystems.

The term “polycrisis” describes situations where multiple crises interact such that the overall impact exceeds the sum of individual shocks (European Commission 2023; World Economic Forum 2023). The co‐occurrence of interconnected shocks—ranging from pandemics and energy disruptions to geopolitical conflicts and climate‐related hazards—has renewed attention to systemic risk (Homer‐Dixon et al. 2015; Lawrence et al. 2024; Tooze 2022; Centeno et al. 2015). In polycrisis contexts, one perturbation can cascade across sectors and borders, amplifying vulnerabilities and overwhelming adaptive capacities. Measuring resilience therefore requires models that capture nonlinear dependencies and interaction effects between diverse risk‐related inputs—precisely the domain where GSA provides analytical insight.

### 3.2 Quantitative Resilience Measurement

Over the past decade, efforts to quantify societal resilience have increasingly relied on indicator‐based monitoring frameworks. Examples include the OECD's fragilities framework (OECD 2018), the State Resilience Index published by the Fund for Peace (FFP 2023), and the JRC Resilience Dashboards, which systematically monitor resilience capacities across EU Member States (Benczur et al. 2023a, 2023b). In parallel, perception‐based indices such as the LRF World Risk Poll Resilience Index have emerged to assess resilience as experienced and reported by citizens, complementing structural measures with behavioral and psychological dimensions (Lloyd's Register Foundation 2022, 2024).

Structural dashboards quantify the potential capacity of systems to absorb shocks, whilst perception‐based indices capture the realized or perceived resilience capacity at the societal level. However, the two measures have rarely been integrated analytically. Their coexistence highlights a central methodological gap: although each provides information, neither alone reveals how structural capacities translate into resilience outcomes. To bridge this gap, a modeling framework is needed capable of handling interdependencies, nonlinearities, and shared variance among indicators.

### 3.3 Sensitivity Analysis in Risk Assessment and the Shapley Solution

SA is a core component of quantitative risk assessment, aiming to understand how uncertainty or variability in model inputs propagates to uncertainty in model outputs (Saltelli et al. 2008; Borgonovo 2017). Local SA approaches, such as one‐at‐a‐time perturbations, assess marginal effects around a reference point but are inadequate for complex systems, as they fail to capture nonlinear responses, interaction effects, and joint input variation (Saltelli et al. 2008; Helton et al. 2006). GSA addresses these challenges by evaluating the entire input space and decomposing output variability into contributions attributable to individual inputs and their interactions (Anderson et al. 2014; Mara and Tarantola 2012).

Classical variance‐based methods, most notably Sobol’ indices, partition total output variance into first‐order and higher‐order components (Sobol’ 2001; Saltelli et al. 2010). However, Sobol’‐type decompositions rely on an assumption of input independence that is rarely satisfied in real‐world socioeconomic and policy systems. Under such dependence, classical variance‐based indices can become biased and may misattribute importance across inputs (Borgonovo et al. 2012; Owen and Prieur 2017).

To overcome these limitations, Shapley‐value‐based SA has been proposed as a principled generalization of variance decomposition under input dependence. Originating in cooperative game theory (Shapley 1953), the Shapley framework treats each input variable as a player contributing to overall model performance. The Shapley value of an input is defined as its average marginal contribution across all possible coalitions of inputs, ensuring fairness, efficiency, and symmetry in attribution (Hart and Mas‐Colell 1989; Owen 2014). Crucially, Shapley effects remain well‐defined under correlated inputs and nonlinear interactions, making them particularly suitable for societal and policy‐oriented models.

A key practical challenge in applying Shapley‐based GSA is the computational burden associated with evaluating model performance across a large number of input coalitions, particularly in nonlinear settings or when sample size is limited. Surrogate modeling addresses this challenge by approximating the input–output relationship in a computationally efficient and numerically stable manner. The use of metamodels—including Gaussian processes and PCE—for Shapley effect estimation was first formalized by Iooss and Prieur (2019), who showed that surrogate‐based approaches can provide consistent and interpretable Shapley decompositions under correlated inputs. Building on this line of work, Torre et al. (2022) and Lüthen et al. (2023) developed PCE‐specific methods for estimating Shapley effects and SHAP values, respectively, demonstrating that the polynomial coefficient structure of a fitted PCE surrogate permits analytical derivation of variance‐based sensitivity metrics. Among surrogate techniques, PCE represents the model output as an expansion over orthogonal polynomial basis functions (Xiu and Karniadakis 2002; Sudret 2008; Shao et al. 2017; Mara and Becker 2021), allowing nonlinear effects and interaction structures to be captured through polynomial terms. When combined with regularization and sparse basis selection, PCE can provide accurate surrogate representations in low‐dimensional settings with limited sample size, while retaining analytical tractability.

It is important to note that Shapley effects should not be interpreted as a direct extension of classical variance decomposition: they arise from a distinct allocation mechanism governed by the game‐theoretic axioms of efficiency, symmetry, and additivity, which classical Sobol’ indices do not satisfy under input dependence (Owen 2014; Plischke et al. 2021). Nonetheless, when computed on PCE surrogates, Shapley values provide consistent and interpretable decompositions of model explanatory power expressed on a variance‐explained scale comparable to variance‐based sensitivity metrics, thereby bridging classical GSA with modern feature attribution frameworks (Borgonovo and Plischke 2016; Razavi et al. 2021).

Despite the growing methodological maturity of Shapley‐based SA and surrogate modeling, these approaches remain largely absent from empirical societal resilience research, particularly in small‐sample comparative settings characterized by strongly correlated structural indicators. In this context, the contribution of the present study is not to propose a new estimator but to adapt and integrate established GSA tools into a coherent analytical workflow suitable for cross‐national resilience assessment.

By combining conservative screening, stability‐based variable selection, PCE surrogate modeling, and Shapley‐based variance attribution, the study operationalizes a framework capable of estimating structurally relevant resilience‐related contributions under conditions where classical regression‐based attribution becomes unstable. This allows established SA principles to be transferred into a policy‐relevant resilience setting where methodological transparency and interpretability are particularly important.

## 4 Data and Methodology

### 4.1 Data

#### 4.1.1 Dependent Variable: Lloyd's Resilience Index

Our dependent variable is a national‐level proxy for societal resilience measured through the LRF World Risk Poll Resilience Index. The index conceptualizes resilience as the perceived and experienced ability of individuals and communities to recover from difficulties, adapt to changing conditions, and maintain well‐being in the face of shocks and stressors (Lloyd's Register Foundation 2022). Importantly, this is an outcome‐oriented and perception‐based measure. The index is derived from biennial Gallup surveys covering more than 125,000 respondents across 142 countries and captures resilience across four interlinked levels: individual, household, community, and societal. Methodologically, the index is constructed through a latent‐variable scaling approach applied to survey items related to trust, preparedness, social support, and institutional effectiveness. Scores are standardized on a 0–100 scale, with higher values indicating greater perceived resilience. In this study, we use the societal‐level resilience score as the national‐level outcome variable. Whereas structural indicators capture institutional and socioeconomic capacities, the LRF index reflects how such capacities are experienced and translated into perceived resilience outcomes, making it a suitable dependent variable for variance‐based attribution under conditions of interdependence and nonlinearity.

#### 4.1.2 Independent Variables: JRC Resilience Dashboard

Our independent variables are drawn from the JRC Resilience Dashboards, a harmonized monitoring framework designed to capture the structural capacities underpinning societal resilience across EU Member States. The Dashboard comprises 124 quantitative indicators compiled from authoritative international sources, including Eurostat, Organisation for Economic Cooperation and Development, and World Bank (Benczur et al. 2023a, 2023b), organized into four analytical dimensions: socioeconomic, green, digital, and geopolitical. The socioeconomic dimension captures labor‐market conditions, income distribution, education and skills, and institutional quality. The green dimension reflects environmental sustainability and ecological transition capacity. The digital dimension measures technological preparedness and digital inclusion. The geopolitical dimension captures strategic exposure, external dependencies, and aspects of strategic autonomy.

The use of two independent databases is analytically deliberate: the JRC Resilience Dashboard captures structural resilience capacities, whereas the LRF Resilience Index captures realized resilience outcomes based on independently collected survey evidence. Their combination allows structural indicators and resilience outcomes to be analyzed jointly while reducing the risk of conceptual and statistical circularity.

#### 4.1.3 Data Harmonization

All JRC indicators are provided in standardized and directionally aligned form, such that higher values consistently correspond to greater structural resilience capacity (Benczur et al. 2023a, 2023b). Indicators with missing values for more than three EU Member States were excluded to preserve cross‐national comparability. Remaining missing observations (less than 5% of the total) were imputed using mean substitution within EU subregions (North, South, East, and West).

Both datasets were aligned into cross‐sectional country–year analytical matrices covering the 27 EU Member States for 2021 and 2023, yielding two analytical datasets of *n* = 27 observations by 124 candidate indicators. The EU‐27 average is retained only for descriptive comparison in selected figures and is not included in model estimation.

### 4.2 Methodology

#### 4.2.1 Shapley‐Based Sensitivity Analysis: Overview and Rationale

To identify the structural indicators most strongly associated with societal resilience, we implement a multistage analytical framework operating in a given data context (*n* = 27 EU Member States, *p* = 124 candidate indicators). Throughout the article, “small‐n” refers to this limited cross‐national sample size (*n* = 27), which constrains model complexity and motivates conservative screening and validation choices.

The choice of Shapley effects is motivated primarily by the presence of substantial predictor correlation, which makes variance attribution through independence‐based sensitivity measures difficult to interpret. Whereas first‐order Sobol’ indices rely on independence assumptions, Shapley effects remain well‐defined under dependence by averaging marginal contributions across all possible predictor coalitions. This makes Shapley decomposition particularly suitable for resilience datasets, where structural indicators frequently overlap both conceptually and statistically.

The analytical design addresses three challenges inherent in cross‐national resilience modeling: high dimensionality, correlated predictors, and limited sample size. As a first preprocessing step, the initial pool of 124 indicators is reduced using univariate Pearson correlation screening at a nominal significance level (*p* ≤ 0.05), retaining indicators exhibiting statistically significant associations with the Lloyd's Resilience Index. This reduced the candidate pool to 43 indicators for 2021 and 38 indicators for 2023. To address redundancy and improve robustness under correlated inputs, RF‐based stability selection is then applied using a selection frequency threshold of 0.55 over 500 resampling iterations (top*‐k* = 10 indicators per iteration), yielding a final set of *d* = 8 indicators for 2021 and *d* = 10 indicators for 2023. The threshold of 0.55 was chosen to retain variables showing majority‐level selection consistency while avoiding excessive exclusion under small‐sample conditions.

Finally, the explanatory variance captured by a PCE regression metamodel fitted to these selected indicators is decomposed using Shapley‐based GSA, yielding an additive and Shapley‐consistent attribution of model performance across indicators. Here, *d* denotes the reduced predictor dimension entering the surrogate model and therefore differs from both the initial candidate space (*p* = 124) and the observation count (*n* = 27).

#### 4.2.2 Variable Assessment and Initial Screening

The JRC Resilience Dashboards comprise 124 indicators spanning socioeconomic, green, digital, and geopolitical dimensions. Each indicator $X_{j}$ is first assessed individually using the Pearson correlation coefficient with the Lloyd's Resilience Index $Y_{i}\left(i=1,\text{…},n;n=27\right)$. This approach is consistent with SA screening practice (Saltelli et al. 2008; Helton et al. 2006) and provides a transparent first‐stage assessment under severe dimensionality constraints.

This reduction stage serves a preparatory rather than inferential role: its primary purpose is to stabilize dimensionality before surrogate‐based variance attribution becomes feasible. Statistical significance is initially evaluated at nominal *p* ≤ 0.05, and indicators meeting this criterion are retained as candidate variables. Because this stage is exploratory rather than inferential, the nominal threshold is therefore used only to reduce dimensionality prior to stability‐based multivariate selection, not to support standalone inferential claims. Potential false positives are subsequently filtered through RF stability selection, which retains only variables showing consistent importance under repeated resampling.

Whilst more sophisticated screening techniques exist, including the Hilbert–Schmidt Independence Criterion (HSIC) for detecting nonlinear dependence (De Lozzo and Marrel 2016) and Johnson relative weights for correlated multilinear settings (Johnson 2000; Clouvel et al. 2025), Pearson correlation is preferred here because the small sample size (*n* = 27) limits the stability of kernel‐based methods, while the objective of this stage is conservative dimensionality reduction rather than nonlinear dependency characterization.

This screening step yielded 43 candidate indicators for 2021 and 38 candidate indicators for 2023.

#### 4.2.3 Indicator Selection via RF Stability Analysis

Final variable retention does not depend on Pearson correlation alone, but on repeated RF selection frequencies across resampled training sets. The RF procedure is used here as a stability‐oriented ranking device rather than as a predictive estimator, with repeated resampling intended to identify variables that remain consistently important under moderate perturbations of the sample.

Following univariate screening, indicator selection is refined using an RF stability selection procedure (Breiman 2001; Meinshausen and Bühlmann 2010). For each reference year, an RF regressor is repeatedly fitted on random subsamples of countries comprising 80% of observations drawn without replacement. This subsampling fraction balances sample perturbation with sufficient retained observations for stable estimation in the *n* = 27 setting.

In each iteration, permutation importance scores are computed for all candidate indicators, and only the top‐*k* = 10 highest‐ranked indicators are retained. Here, top‐*k* denotes the fixed number of variables retained per iteration before aggregation of selection frequencies across repetitions.

This procedure is repeated 500 times, yielding for each indicator a selection frequency defined as the proportion of iterations in which it appears among the top 10. Indicators with selection frequencies exceeding 0.55 are retained for subsequent analysis. The number of repetitions was chosen to ensure stable frequency estimates without unnecessary computational expansion, given the reduced dimensionality after prescreening. This stability‐based procedure prioritizes indicators displaying consistent predictive relevance across resampled datasets, an important safeguard under small‐sample and correlated‐input conditions.

The final retained predictor set consists of *d* = 8 indicators for 2021 and *d* = 10 indicators for 2023. Although RF‐based Shapley estimation has been developed in the literature (Bénard et al. 2022), RF is used here exclusively for stability‐based screening rather than for Shapley computation. Shapley decomposition is instead performed on a PCE surrogate, whose polynomial coefficient structure permits analytical variance decomposition and efficient estimation under correlated inputs (Mara and Becker 2021; Iooss and Prieur 2019; Torre et al. 2022). This stage therefore addresses variable stability rather than variance attribution and defines the predictor space entering the surrogate model.

#### 4.2.4 PCE Surrogate Model

Whereas RF is used for stability‐based ranking, PCE is introduced only after dimensionality reduction in order to approximate the empirical response surface required for Shapley decomposition. The two stages therefore address complementary analytical problems: dimensional feasibility first, variance attribution second.

To enable efficient and numerically stable computation of Shapley values while allowing for nonlinear effects and interactions, the mapping f:X→Y is approximated using a PCE surrogate model (Blatman and Sudret 2011; Shao et al. 2017; Mara and Becker 2021). Unlike classical given‐model SA, the surrogate here is estimated directly from observed country‐level data and serves to approximate the empirical response surface required for variance decomposition.

Given the limited sample size, the polynomial basis is deliberately kept low‐order so that the dominant variance structure can be approximated without generating excessive coefficient complexity relative to available observations. The surrogate is therefore specified conservatively, prioritizing stability of variance attribution over maximal functional flexibility.

Prior to PCE fitting, predictor variables are internally z‐standardized (mean 0, variance 1). Extreme values are clipped using z‐score clipping (±6) to prevent numerical instabilities during polynomial construction. The target variable $Y_{i}$ is standardized as

$$\;y_{s,i}=\frac{y_{i}-\overset{\prime }{y}}{\sigma_{y}},$$

where $\overset{\prime }{y}$ denotes the sample mean and $\sigma_{y}$ denotes the sample standard deviation.

The PCE basis is constructed using a sparse multi‐index set constrained by (i) a maximum total polynomial degree, automatically reduced for small sample sizes, and (ii) a maximum interaction order of two, allowing pairwise interactions by default and reduced further when sample size is limited. Model coefficients are estimated via a ridge‐regularized least‐squares solution:

$$\hat{\beta }=argmin_{\beta }∥y_{s}-\Psi \left(X\right)\beta {∥}^{2}+\lambda ∥\beta {∥}^{2},$$

(the notation therefore refers to a minimizing solution rather than a unique estimator), where β is the vector of unknown PCE coefficients, $\hat{\beta \;}$ denotes a solution minimizing the penalized objective function, X is the matrix of standardized predictors, Ψ(X) is the PCE design matrix (n×K), $∥y_{s}-\Psi \left(X\right)\beta {∥}^{2}$ is the residual sum of squares, $∥\beta \;{∥}^{2}=\sum \beta_{k}^{2}$ penalizes large coefficients, and $\lambda =10^{-6}$ controls regularization strength.

For 2021, the fitted PCE comprised *K* = 14 basis terms; for 2023, *K* = 18 basis terms. The surrogate coefficients are estimated over the reduced predictor space only.

Model fit is evaluated using the coefficient of determination $R^{2}$ computed via LOO cross‐validation, yielding $R^{2}=0.82$ for 2021 and $R^{2}=0.85$ for 2023. These values are interpreted as sufficient surrogate fidelity for variance attribution rather than predictive optimization, as the objective of the PCE approximation is to stabilize Shapley decomposition under correlated small‐sample conditions rather than maximize predictive accuracy. The remaining unexplained variance is retained as substantively meaningful, likely reflecting latent resilience factors, measurement uncertainty, and cross‐country heterogeneity not captured by the selected indicators.

Model performance for Shapley decomposition is additionally summarized through the mean squared residual error:

$$\mathrm{Residual}=E\left[{\left(y_{s}-{\hat{y}}_{s}\right)}^{2}\right],$$

which captures surrogate approximation error and forms the basis for defining the payoff function in the subsequent section.

#### 4.2.5 Shapley Value Decomposition

Conceptually, the approach follows the cooperative‐game‐theoretic principle that total explained variance is treated as a value to be allocated across predictors according to their average marginal contributions across all possible coalitions. This preserves the efficiency, symmetry, and additivity properties of Shapley allocation while adapting them to variance‐based SA.

The *d* indicators retained through stability selection define the player set N={1,…,d} used in the Shapley decomposition. For any coalition S⊆N, the payoff function is defined as

$$v\left(S\right)\;=\max\left(0,1-\mathrm{Residual}\left(S\right)\right),$$

where Residual(*S*) denotes the mean squared error of a PCE surrogate fitted using only the indicators in coalition S. Because the target variable is standardized to unit variance, 1−Residual(S) corresponds to the proportion of explained variance attributable to coalition S. The truncation at zero prevents negative payoff values when coalition‐specific surrogate fits are unstable in small samples.

This payoff function therefore maps surrogate fit into a bounded explained‐variance score consistent with the value‐function framework proposed by Song et al. (2016). Input variables are treated as random variables following their empirical marginal distributions estimated from the observed cross‐national data. Under this empirical design, no parametric distributional assumption is imposed; instead, inputs are standardized empirically, and coalition‐specific PCE surrogates are estimated directly from the observed sample.

The Shapley value for indicator i∈N is given by (Shapley (1953); Owen (2014)):

$$\phi_{i}=\sum_{S\subseteq N\setminus \left\{i\right\}}\frac{|S|!\left(d-|S|-1\right)!}{d!}\;\left[v\left(S\cup \left\{i\right\}\right)-v\left(S\right)\right].$$

This formulation, equivalent to the characterization proposed by Myerson (1991) and implementable through the path‐independent algorithm of Plischke et al. (2021), represents the average marginal contribution of indicator i across all predictor coalitions, satisfying efficiency, symmetry, and additivity.

This logic is closely related to regression‐based LMG decomposition, which also averages marginal $R^{2}$ contributions across predictor orderings. The present framework differs in that variance attribution is performed on a surrogate response surface rather than directly within a linear regression model, allowing Shapley decomposition to remain applicable when nonlinear response structure and correlated predictors are both relevant.

Because exact enumeration remains computationally feasible for both retained predictor sets (2^8^ = 256 coalitions for 2021; 2^10^ = 1024 coalitions for 2023), exact Shapley values are computed for both years using full coalition enumeration.

Because the number of retained predictors remains small relative to the original candidate space, exact coalition enumeration is computationally trivial after screening, eliminating approximation error at the attribution stage.

## 5 Application: Variance Decomposition and Feature Contributions

### 5.1 Screening Results and Indicator Correlation Structure

Univariate Pearson correlation screening (nominal *p* ≤ 0.05) reduced the initial pool of 124 indicators to 43 candidates for 2021 and 38 candidates for 2023. Pearson correlation is used here only as a transparent first‐stage filter and does not constitute the main variable importance criterion of the analysis. Potential nonlinear relationships and interactions are subsequently addressed through RF stability selection and surrogate‐based variance decomposition. Under *n* = 27, more complex dependence estimators may become unstable or overly sensitive to sample perturbation, which further supports the use of a conservative first‐stage filter.

RF stability selection (500 iterations, top‐*k* = 10, threshold = 0.55) then identified *d* = 8 indicators for 2021 and *d* = 10 indicators for 2023 as the final analytical sets. This first‐stage screening is intended as dimensionality reduction under small‐sample conditions rather than as a substantive exclusion rule, removing variables with minimal direct empirical association before stability‐based multivariate selection. The Pearson correlations of selected indicators with the Resilience Index ranged from |*r*| = 0.52 to |*r*| = 0.79 for 2021 and from |*r*| = 0.55 to |*r*| = 0.83 for 2023. The threshold is applied uniformly across all indicators to ensure reproducibility and to avoid discretionary preselection.

Pairwise correlations among the selected indicators confirm the presence of moderate multicollinearity within each year‐specific model. For 2021, pairwise Pearson correlations among the eight selected indicators range from *r* = −0.12 to *r* = 0.61, with a mean absolute correlation of 0.34. The human‐capital and labor‐market cluster—environment technology patents, active labor market policies, and adult competences—exhibits the highest internal correlations (*r* ≈ 0.45 to 0.61). The digitalization cluster—telework constraints, e‐government non‐users, cybersecurity awareness, and judicial system e‐tools—shows moderate intercorrelations (*r* ≈ 0.30 to 0.52). Active citizenship is largely uncorrelated with the remaining indicators (|*r*| ≤ 0.22).

For 2023, pairwise correlations range from *r* = −0.08 to *r* = 0.67, with a mean absolute correlation of 0.38. Antimicrobial resistance and years of life lost due to PM_2_._5_ exposure are moderately correlated with regional dispersion in household income (*r* ≈ 0.51 to 0.58), consistent with the clustering patterns discussed in Section 5.3. These moderate correlations confirm the relevance of Shapley‐based attribution rather than classical Sobol’ indices for decomposition under dependent inputs; fully correlated predictors would receive identical Shapley values, preventing misleading attribution in degenerate cases (Borgonovo et al. 2012; Owen and Prieur 2017).

Variance inflation factors ranged from 1.3 to 3.8 for 2021 and from 1.4 to 4.1 for 2023, confirming that multicollinearity, while present, remains below levels that would render individual Shapley attributions unstable.

### 5.2 PCE Surrogate Fit and Shapley Decomposition Results

**FIGURE 1:**
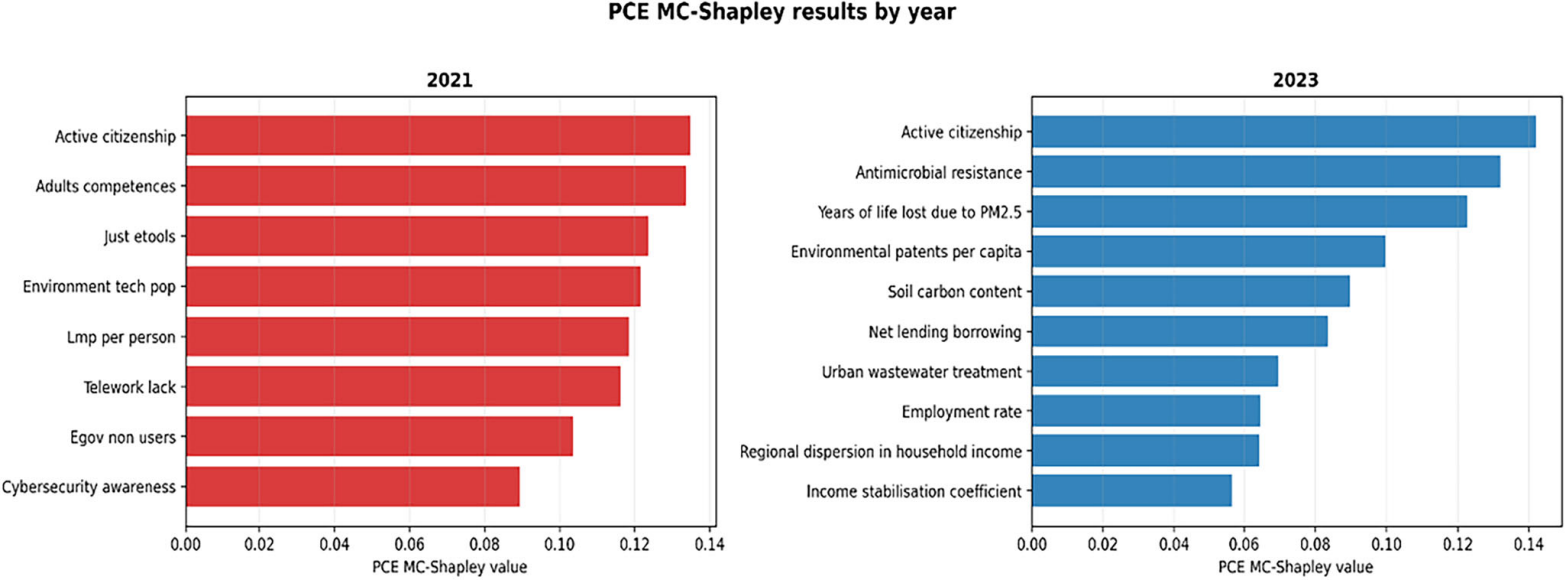
PCE‐MC‐Shapley variance attribution for selected indicators in 2021 and 2023. Bars show absolute Shapley values, interpreted as shares of explained variance from the PCE surrogate models; higher values indicate larger model‐based contributions, not causal effects. Abbreviations: Just e‐tools = judicial system e‐tools; Lmp per person = active labor‐market policies per person; Egov non‐users = individuals not using e‐government services; PM2.5 = fine particulate matter exposure.

Figure 1 reports the PCE‐based Shapley decomposition of the regression metamodel for 2021 and 2023, showing how explained variance in societal resilience scores is allocated across selected structural indicators in each year. The horizontal bars report absolute Shapley values, interpreted as each indicator's share of the fitted model's explanatory capacity after accounting for correlations, nonlinearities, and interaction effects. The PCE surrogate for 2021 comprised *K* = 14 basis terms and achieved *R* ^2^ = 0.82 (evaluated by LOO cross‐validation); for 2023, the surrogate comprised *K* = 18 basis terms and achieved *R* ^2^ = 0.85, likewise evaluated by LOO cross‐validation.

For 2021, the Shapley decomposition reveals a comparatively diffuse resilience structure, with variance attribution distributed across a broad set of indicators. These percentages should be interpreted as relative variance contributions within the fitted surrogate model rather than as causal effect sizes. Active citizenship (ϕ=0.135) and adult competences (ϕ=0.134) have the largest model‐based contributions, followed by judicial system e‐tools (ϕ=0.124) and environmental technology patents per capita (ϕ=0.122). Additional contributions stem from active labor‐market policies per person (ϕ=0.119) and telework constraints (ϕ=0.116), while e‐government non‐users (ϕ=0.104) and cybersecurity awareness (ϕ=0.089) account for smaller but still meaningful shares of explanatory power. The relatively flat profile of Shapley values indicates that no single indicator dominates the fitted attribution profile; rather, modeled resilience scores in the immediate post‐pandemic context are associated with a broad combination of civic engagement, human capital, labor‐market capacity, and digital governance readiness.

Because Shapley decomposition allocates explained variance rather than identifying causal mechanisms, substantive interpretation remains conditional on the modeling framework and the selected indicator space.

For 2023, the decomposition shows a more concentrated variance structure, consistent with the higher parsimony and explanatory power of the fitted metamodel (*R* ^2^ = 0.85). Active citizenship (ϕ=0.142) has the largest model‐based contribution, followed by antimicrobial resistance (ϕ=0.132) and years of life lost due to PM_2_._5_ exposure (ϕ=0.123). A second tier of indicators includes environmental patents per capita (ϕ=0.100) and soil carbon content (ϕ=0.090), suggesting a comparatively strong explanatory role for environmental innovation and ecosystem conditions. Macroeconomic and infrastructural factors—net lending/borrowing (ϕ=0.083), urban wastewater treatment (ϕ=0.070), and employment rate (ϕ=0.065)—provide additional explanatory power, while regional dispersion in household income (ϕ=0.064) and the income stabilization coefficient (ϕ=0.057) contribute more modestly at the margin. The sharper decline in Shapley values beyond the top contributors indicates diminishing marginal explanatory returns from additional indicators, suggesting that, within the fitted model, resilience scores under sustained polycrisis conditions are more closely associated with a narrower set of structural indicators spanning social capital, environmental health, and macroeconomic stabilization capacity.

Overall, the relative variance structure differs clearly between 2021 and 2023. The comparison indicates that the 2023 model achieves slightly higher explanatory power with a more concentrated attribution profile, whereas the 2021 model distributes explanatory variance more evenly across indicators. Because only two cross‐sectional observations are compared, these differences should be interpreted as structured contrasts between resilience contexts rather than evidence of continuous temporal dynamics. The contrast suggests a shift in the fitted attribution profile: whereas resilience scores in 2021 were associated with a broader set of recovery‐related capacities, by 2023 the attribution was concentrated more clearly around persistent structural constraints, particularly environmental health, public health robustness, and civic engagement.

The benchmark LMG decomposition produced similar rankings for the top contributors, but PCE‐Shapley yielded stronger differentiation among mid‐ranking indicators, indicating that interaction effects may influence attribution within the fitted model.

### 5.3 Country‐Level Feature Contributions and Indicator Clustering

**FIGURE 2:**
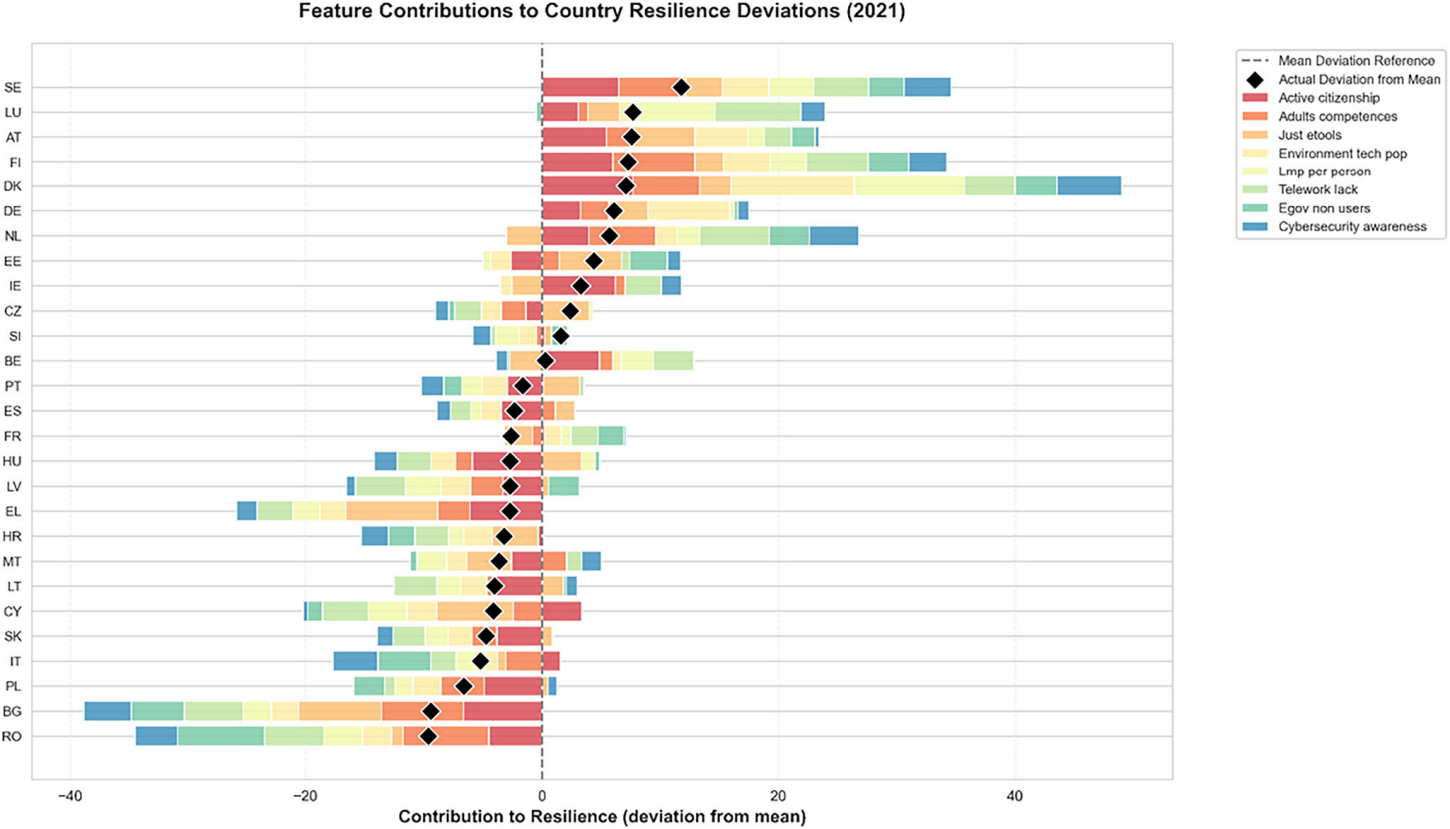
Country‐level decomposition of deviations from the EU‐average resilience score, 2021. Stacked bars show additive Shapley‐based feature contributions by indicator; the dashed vertical line marks zero deviation from the EU average, and black diamonds show each country's observed deviation from the mean. Country codes refer to EU Member States.

Figure 2 decomposes each country's deviation from the EU‐average resilience score in 2021 into additive Shapley‐based feature contributions. Countries positioned to the right of the zero‐reference line have above‐average observed scores associated with the cumulative contribution of several moderately positive indicators rather than reliance on a single dominant factor. Countries to the left have below‐average observed scores associated with reinforcing negative contributions across multiple dimensions. The absence of strong one‐dimensional compensation effects suggests that resilience in 2021 behaved, within the fitted model, as a multi‐constraint outcome: deficits in one domain were not fully offset by strengths in another.

**FIGURE 3:**
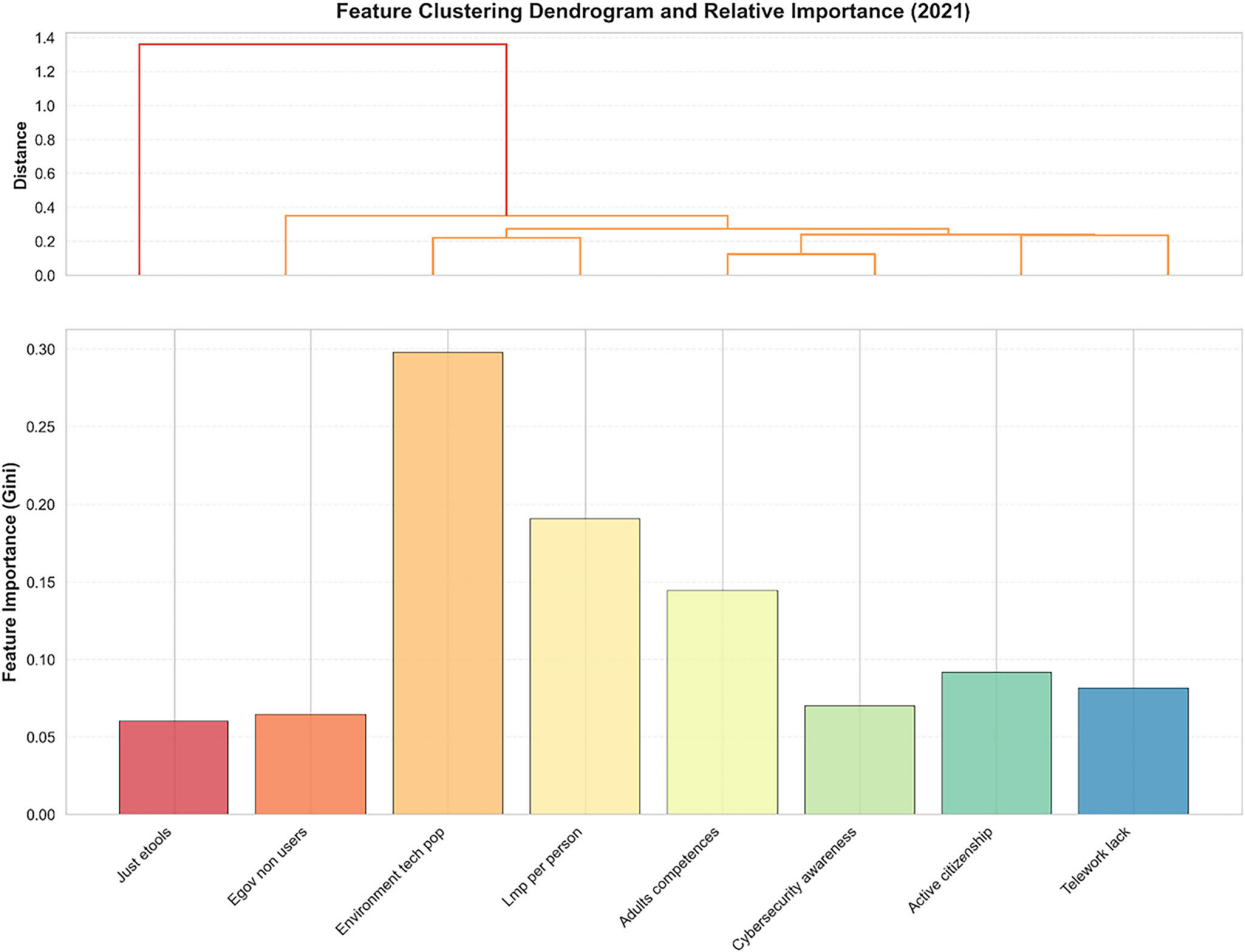
Indicator clustering and relative importance, 2021. The upper dendrogram groups indicators by similarity in country‐level Shapley contribution profiles using Ward‐linkage hierarchical clustering on Euclidean distances; the lower bars report Gini‐style relative importance. Short labels correspond to the indicator names used in Figure 1.

Figure 3 combines information on the relative importance of indicators with their degree of overlap in explaining societal resilience scores in 2021. The dendrogram (constructed via Ward‐linkage hierarchical clustering on Euclidean distances between country‐level Shapley contribution vectors) gathers indicators according to how similarly they contribute to the fitted country‐level decomposition across EU Member States. A central group of labor‐market and human‐capital indicators—environmental technology patents, active labor‐market policies, and adult competences—displays closely related contribution patterns during the post‐pandemic recovery. A second group comprises digitalization‐related indicators, whilst active citizenship appears more weakly connected to the other indicators, highlighting its distinct model‐based contribution beyond labor‐market and digital dimensions.

**FIGURE 4:**
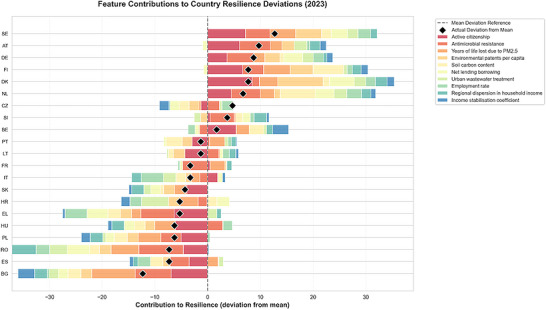
Country‐level decomposition of deviations from the EU‐average resilience score, 2023. Stacked bars show additive Shapley‐based feature contributions by indicator; the dashed vertical line marks zero deviation from the EU average, and black diamonds show each country's observed deviation from the mean. Positive and negative segments indicate model‐based contributions above or below the EU‐average profile.

Figure 4 decomposes each country's deviation from the EU‐average resilience score in 2023 into additive Shapley‐based feature contributions. Compared with 2021, the contribution patterns appear more structured and less dispersed across countries. Positive deviations from the EU average are typically associated with positive model‐based contributions from active citizenship and environmental and public health indicators, whilst negative deviations are mainly associated with negative contributions from PM_2_._5_‐related mortality and antimicrobial resistance.

**FIGURE 5:**
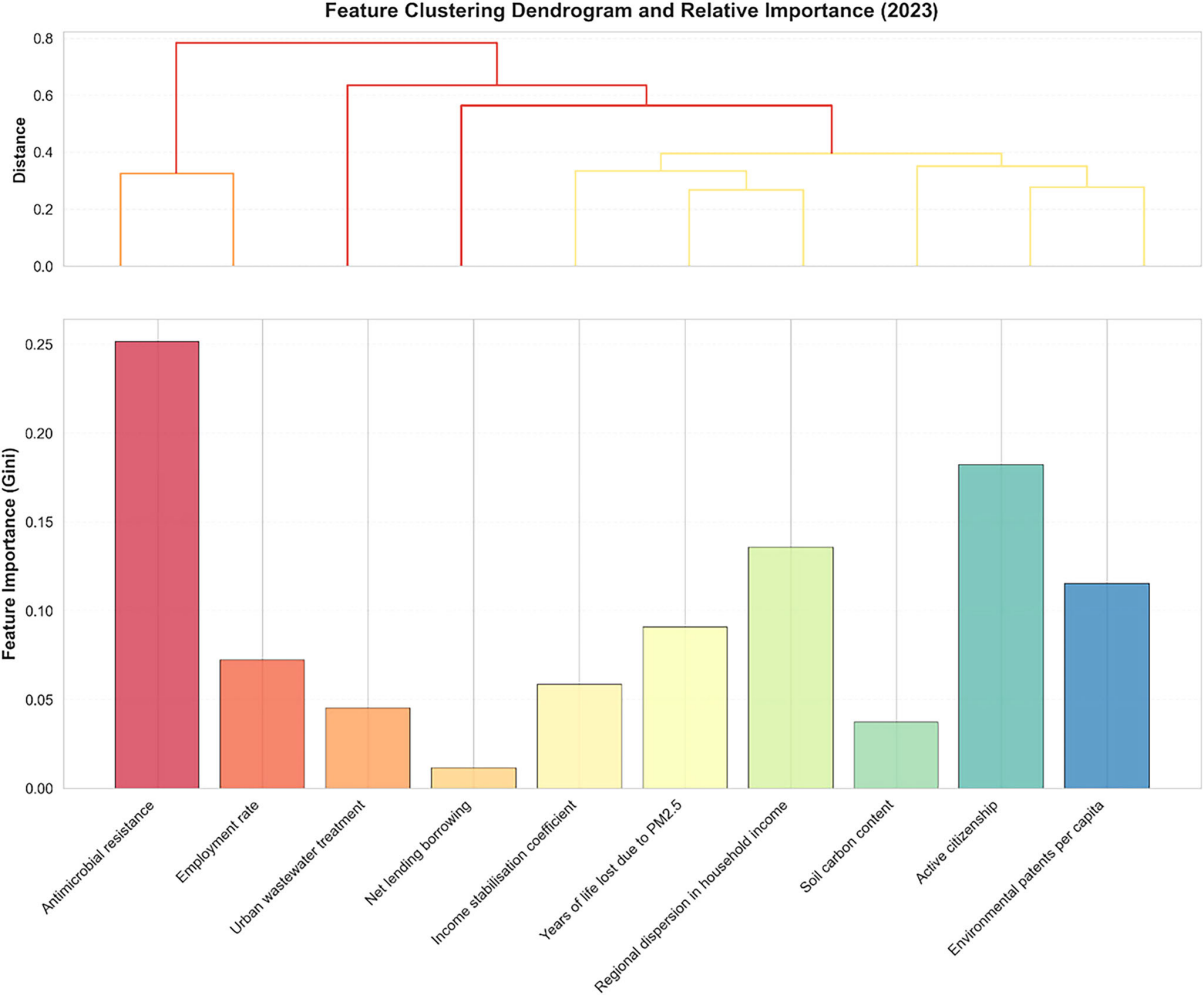
Indicator clustering and relative importance, 2023. The dendrogram groups indicators by similarity in country‐level Shapley contribution profiles using Ward‐linkage hierarchical clustering on Euclidean distances; lower bars report Gini‐style relative importance. Clusters describe co‐varying attribution profiles rather than causal pathways.

Figure 5 presents indicator clustering for 2023. Clustering is used here descriptively to identify groups of indicators whose country‐level contribution profiles co‐vary across Member States. The dendrogram shows a clearly defined cluster centered on health, environmental quality, and distributional structure. Antimicrobial resistance and years of life lost due to PM_2_._5_ exposure merge at relatively low distances with regional dispersion in household income and soil carbon content, indicating that public health, environmental, and inequality‐related contribution profiles tend to co‐vary under sustained polycrisis conditions. A second, more compact cluster comprises macroeconomic buffering, infrastructure, and innovation capacity indicators. Active citizenship joins the dendrogram at a higher linkage distance, highlighting a contribution pattern not tightly aligned with either health‐environmental constraints or macroeconomic adjustment mechanisms.

## 6 Robustness and Residual Uncertainty: LOO Analysis of Shapley Attributions

To assess the sensitivity of the Shapley‐based variance attributions to influential observations, we implement a LOO robustness analysis (Kohavi 1995; Vehtari et al. 2017). Given the limited sample size inherent in cross‐national EU analysis (*n* = 27 Member States), this procedure evaluates whether estimated indicator contributions are strongly affected by individual country exclusions. The LOO analysis is conducted separately for 2021 and 2023, conditional on the final year‐specific indicator sets identified through RF stability selection.

For each year, the LOO procedure iteratively removes one country from the dataset and recomputes the full Shapley decomposition on the remaining *n* − 1 observations. The indicator set is held fixed across folds, ensuring that variation in Shapley values reflects sensitivity to country exclusion rather than changes in model specification or feature selection. In each fold, the PCE surrogate is refitted, and exact Shapley values are recomputed using full coalition enumeration. This yields, for each indicator, an empirical distribution of Shapley values across the 27 LOO iterations. This exercise provides a useful empirical robustness check, but it does not yield formal confidence intervals or uncertainty bands for the Shapley estimates; residual uncertainty therefore remains substantial in a sample of 27 countries.

The resulting LOO Shapley distributions are analyzed using kernel density estimates, allowing visual assessment of attribution stability within and across years. Narrow, unimodal distributions indicate indicators whose contributions are relatively stable under country exclusion, whereas wider or skewed distributions signal greater sensitivity to specific country contexts. Comparing LOO distributions between 2021 and 2023, using the union of year‐specific indicator sets, further distinguishes indicators with recurring explanatory roles from those whose importance is more contingent on period‐specific resilience structures.

**FIGURE 6:**
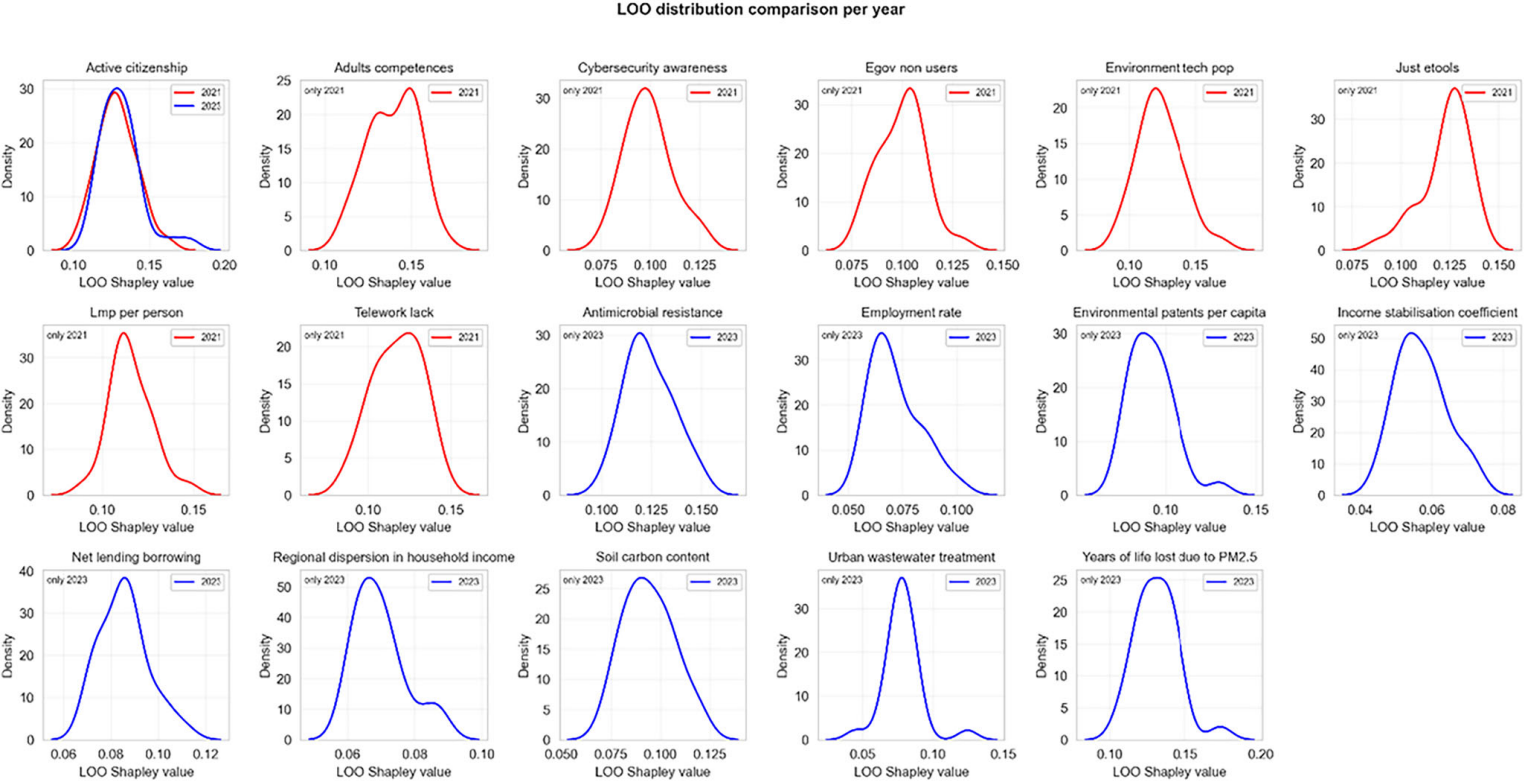
LOO distributions of Shapley values by year. Kernel density curves summarize the empirical distribution of each indicator's Shapley attribution after excluding one EU Member State at a time. Red curves denote 2021; blue curves denote 2023; panels marked “only 2021” or “only 2023” indicate indicators selected in only one year.

Figure 6 presents the LOO distributions of Shapley values for the final indicators selected in each year. Across both years, the distributions are generally narrow and unimodal, suggesting that Shapley attributions are not highly sensitive to individual country exclusions. The only indicator common to both years, active citizenship, exhibits highly overlapping LOO distributions in 2021 and 2023, with similar central tendencies and dispersion, suggesting a stable model‐based contribution to societal resilience scores across both analytical contexts.

Indicators selected only in 2021 also display relatively high stability. Adult competences, cybersecurity awareness, e‐government non‐users, environmental technology patents, judicial system e‐tools, and active labor‐market policies per person all show unimodal distributions with limited dispersion. Telework constraints exhibit a somewhat wider distribution with mild asymmetry, reflecting heterogeneous labor‐market and remote‐work conditions across Member States; however, the absence of multimodality suggests limited outlier dependence rather than full certainty.

Indicators selected only in 2023 show similar patterns. Antimicrobial resistance and years of life lost due to PM_2_._5_ exposure display concentrated unimodal distributions, suggesting stable model‐based contributions with moderate cross‐national heterogeneity. Environmental indicators—environmental patents per capita and soil carbon content—are likewise stable. Infrastructural and macroeconomic indicators show comparatively tight distributions consistent with less country‐specific sensitivity. Employment rate presents a sharp central peak with a longer right tail, suggesting stable average contributions that increase under specific country exclusions.

Overall, the LOO results do not indicate that the Shapley attributions are dominated by single‐country outliers. Differences between 2021 and 2023 appear to reflect variation in the indicator sets identified through stability selection and the year‐specific fitted models, rather than obvious instability in the Shapley decomposition alone. This supports a cautious interpretation of year‐specific attribution patterns, while recognizing that formal uncertainty bands are not estimated.

## 7 Discussion

The contrast between 2021 and 2023 suggests a change in the relative structure of model‐based resilience associations. In 2021, resilience scores were associated with a relatively broad and balanced set of human‐capital, labor‐market, and digitalization indicators. “Active citizenship,” “Adult competences,” “Judicial system e‐tools,” “E‐government non‐users,” and “Cybersecurity awareness” jointly capture capacities related to institutional functionality, digital access, and civic engagement under acute disruption. Labor‐market and work‐organization indicators—active labor‐market policies and telework constraints—reflect capacities linked to employment continuity and economic activity during lockdowns and mobility restrictions. This diffuse contribution structure is consistent with a transitional resilience context, in which observed resilience scores were associated with short‐term adjustment mechanisms, institutional improvisation, and emergency responses as well as deeper structural conditions (European Commission 2022).

By 2023, however, the fitted attribution structure becomes more concentrated. A small core of indicators—most prominently “Active citizenship,” “Antimicrobial resistance,” and “Years of life lost due to PM_2_._5_ exposure”—accounts for the majority of explained variance, with overall explanatory power increasing despite a more parsimonious model (*R* ^2^ = 0.85 in 2023 vs. *R* ^2^ = 0.82 in 2021). A second tier of associated indicators includes “Environmental patents per capita,” “Soil carbon content,” and “Urban wastewater treatment,” capturing longer‐term environmental quality, innovation capacity, and infrastructure robustness (IPCC 2022). Economic stabilization and buffering indicators—net lending/borrowing, employment rate, income stabilization coefficient, and regional dispersion in household income—provide additional explanatory contributions. This more concentrated attribution pattern suggests that, relative to 2021, resilience scores in 2023 were more strongly associated with persistent structural conditions than with short‐term adaptive adjustment mechanisms.

A particularly salient model‐based association concerns social capital. “Active citizenship” has one of the largest Shapley shares in 2023, and its share is larger than in 2021. This pattern is consistent with resilience theory emphasizing collective action, trust, and community engagement as central mechanisms for sustaining adaptive capacity under prolonged stress (Norris et al. 2008; Aldrich and Meyer 2015). Whilst state‐led interventions, digital service continuity, and labor‐market protections were critical during the acute phase of the pandemic, sustained polycrisis is plausibly associated with greater reliance on horizontal, community‐based resilience mechanisms operating beyond formal institutional channels (Aldrich 2012). The LOO results suggest that this association is not driven by a single country, but they do not establish a direct causal effect of civic participation. The result indicates that countries combining stronger civic engagement with other favorable structural conditions tend to exhibit higher explained resilience scores.

Environmental and health‐related indicators are prominent in the post‐pandemic attribution structure. “Years of life lost due to PM_2_._5_ exposure” and “Antimicrobial resistance” together form a dominant cluster, indicating that environmental quality and public health capacity are closely associated with modeled resilience scores (IPCC 2022). Complementary environmental indicators—such as “Soil carbon content,” “Urban wastewater treatment,” and “Environmental patents per capita”—reinforce this interpretation by linking ecosystem health, infrastructure, and innovation capacity to the fitted attribution profile (Rockström et al. 2009). Unlike labor‐market or digital indicators, these factors cannot be rapidly adjusted once crises unfold; instead, they reflect long‐term investment trajectories, governance effectiveness, and institutional preparedness (Stern 2007).

The clustering patterns further indicate a close association between environmental health, inequality‐related variables, and public health outcomes. Indicators such as “Regional dispersion in household income” and the “Income stabilisation coefficient” co‐vary with environmental and health constraints, suggesting reinforcing rather than compensatory attribution profiles (Marmot et al. 2008). In this interpretation, societal resilience is not simply the additive sum of sectoral strengths; it is associated with interdependent structural systems in which weaknesses in key domains may constrain adaptive capacity (Folke et al. 2010).

Economic structure and flexibility also appear more prominent in 2023, but in a different form than during the pandemic period. In 2021, economic resilience was primarily associated with employment continuity and work reorganization (OECD 2020). By contrast, the 2023 results highlight indicators related to macroeconomic buffering and innovation capacity, consistent with a more transformative rather than purely defensive mode of economic resilience (Simmie and Martin 2010; Blanchard et al. 2010).

Beyond the empirical findings, the application illustrates the analytical value of transferring GSA into comparative resilience research. Unlike many established SA applications in engineering or environmental modeling, resilience assessment at the cross‐national level combines high indicator interdependence, limited sample size, and conceptually heterogeneous dimensions that make attribution challenging. In this setting, the combination of stability‐based screening and Shapley‐PCE decomposition provides a transparent way to separate more stable model‐based contributions from artifacts of multicollinearity or sample dependence. The methodological relevance lies not in causal identification but in showing that variance‐based attribution can remain informative even when classical inferential assumptions are difficult to satisfy.

These findings should be interpreted as analytical signals regarding structural resilience sensitivity rather than as direct policy prescriptions, because variance attribution alone cannot determine policy priority independently of institutional and normative considerations.

The LOO analysis provides a useful, though limited, check on whether the identified attribution patterns are dominated by individual countries. Across both years, Shapley attributions remain relatively stable under country exclusion, but this does not remove the substantial uncertainty inherent in a small cross‐sectional sample. From a methodological perspective, the findings suggest the value of combining stability‐based selection with Shapley‐PCE GSA in high‐dimensional, small‐n policy settings. From a policy perspective, the results point to structural foundations that merit attention alongside crisis‐specific interventions, without implying a direct hierarchy of policy priorities.

## 8 Conclusions

This study provides an integrated empirical and methodological assessment of structural indicators associated with societal resilience scores across EU Member States in 2021 and 2023. By combining stability‐based feature selection, regression modeling, and Shapley‐based variance decomposition implemented through a PCE surrogate, the analysis identifies a change in the relative structure of model‐based resilience attributions while advancing a transparent and interpretable framework for comparative risk analysis in small‐sample policy settings.

Empirically, the results suggest that crisis context is associated with changes in the relative importance of resilience‐related indicators, while a limited set of foundational associations remains recurrent across analytical contexts. Labor‐market flexibility and digital access play a prominent role during acute recovery conditions, as observed in 2021 (*R* ^2^ = 0.82), whereas environmental health, public health capacity, and social capital have greater explanatory importance under more sustained structural pressure in 2023 (*R* ^2^ = 0.85). The limited evidence of compensatory effects across domains further suggests that resilience scores are associated with balanced structural capacity across interconnected systems rather than isolated strengths in individual sectors.

Methodologically, the article contributes to SA and systemic risk research by showing how econometric modeling can be combined with game‐theoretically grounded importance attribution under conditions of correlated inputs and limited observations. The proposed two‐stage design combines conservative prescreening, PCE surrogate fitting, and Shapley‐based attribution satisfying efficiency, symmetry, and additivity, while LOO validation provides an empirical robustness check against individual country leverage.

Several limitations remain. The reliance on harmonized cross‐national indicators may mask subnational heterogeneity, and the comparison of two cross‐sections limits causal interpretation and the analysis of longer‐term dynamic feedbacks. In addition, Shapley estimates are reported without formal confidence intervals or uncertainty bands; LOO validation should therefore be interpreted as a robustness check rather than full uncertainty quantification. The use of the LRF Resilience Index as the outcome variable captures perceived and experienced resilience rather than the full multidimensional concept discussed in the broader theoretical literature. The results should therefore be interpreted as accounting for variation in lived resilience outcomes within the fitted modeling framework rather than providing a complete representation of all resilience capacities.

These constraints point to future research directions, including panel extensions, higher‐frequency or subnational data, and hybrid modeling strategies combining interpretable surrogates with more flexible machine‐learning approaches. The proposed framework is therefore best understood not as a definitive model but as a methodological starting point for more comprehensive empirical resilience modeling, offering a scalable and transparent tool for monitoring structural vulnerabilities and supporting integrated resilience analysis in Europe and beyond.

## Supporting information




**Supporting Information**: risa70295‐supp‐0001‐SuppMat.csv

**Supporting Information**: risa70295‐supp‐0002‐SuppMat.csv

**Supporting Information**: risa70295‐supp‐0003‐SuppMat.py
